# Macrovascular Hemodynamics and Peripheral Perfusion in Cardiogenic Shock: Exploring Current Targets and Future Directions

**DOI:** 10.1016/j.jacadv.2025.101964

**Published:** 2025-07-23

**Authors:** Leah B. Kosyakovsky, William B. Earle, Colter Wichern, Carla Boyle, Conrad Macon, Rebecca Mathew, Benjamin Hibbert, Joaquin E. Cigarroa, Jeffrey A. Marbach

**Affiliations:** a Division of Cardiovascular Medicine, Beth Israel Deaconess Medical Center, Boston, Massachusetts, USA; b Division of Cardiology, Massachusetts General Hospital, Boston, Massachusetts, USA; c Division of Cardiovascular Medicine, Knight Cardiovascular Institute, Oregon Health & Science University, Portland, Oregon, USA; d Division of Cardiology, University of Ottawa Heart Institute, Ottawa, Ontario, Canada; e Department of Cardiovascular Medicine, Mayo Clinic, Rochester, Massachusetts, USA

**Keywords:** cardiogenic shock, hemodynamics, outcomes, tissue perfusion

## Abstract

Despite significant advances in care over the past few decades, mortality among patients with cardiogenic shock (CS) remains up to 50%. Given the persistently high mortality, there is an urgent need for both better prognostic tools and treatment strategies. The pathophysiology of CS has major contributions from both macrovascular and microvascular dysfunction, but therapies are titrated toward the more readily measurable metrics (ie, mean arterial pressure, cardiac index, etc) under the assumption that both macrovascular and microvascular dynamics will respond to intervention in tandem. However, emerging evidence suggests that macrovascular and microvascular circulatory functions are not always aligned, particularly in those with critical illness. This review summarizes the significance of different macrovascular and microvascular metrics in CS, drawing from a robust field of evidence to demonstrate the promising role that microvascular tissue perfusion markers play in management of patients with CS and summarize the current understanding of this burgeoning field.

## Highlights

• Cardiogenic shock mortality remains high, and there’s an urgent need to improve prognostication and treatment.
• The pathophysiology of cardiogenic shock is contributed to by both microvascular and macrovascular dysfunction.
• Macrovascular metrics are more readily targetable, but do not fully represent tissue oxygenation and perfusion.
• It is critical to also consider how to incorporate microvascular targets into cardiogenic shock management.

The macrovasculature and microvasculature serve distinct functions to maintain circulation. The macrovascular circulation, consisting of large arteries and veins, acts as a conduit for blood flow from the central circulation to peripheral tissues.^1^ Meanwhile, the microvascular circulation, including arterioles, capillaries, and venules, plays a crucial role in regulating blood flow and facilitating the exchange of nutrients, oxygen, and waste products. The function of these 2 systems is assumed to be closely aligned under normal physiological conditions and in critical illness. Accordingly, alterations in macrovascular hemodynamics, which are easier to quantify, are thought to be mirrored in microvascular tissue perfusion. This principle has been a cornerstone in patient management since the advent of continuous hemodynamic monitoring in the 1960s. As a result, the microvascular system’s varied and specialized functions are frequently overlooked in clinical settings, in favor of more readily apparent and quantifiable macrovascular hemodynamic data.^2^ Consequently, the predominant approach to managing circulatory shock, as recommended by societal guidelines and expert consensus, focuses heavily on interventions aimed at improving macrovascular hemodynamic parameters such as systemic arterial pressure, right atrial pressure, pulmonary capillary wedge pressure, and cardiac output (CO).^3^

However, emerging evidence suggests that microvascular abnormalities can persist despite apparently normal macrovascular parameters, leading to inadequate tissue oxygenation and potential organ failure. The assumption that manipulation of macrovascular parameters will inevitably be reflected in tissue perfusion must be re-examined, as the interplay between these 2 components of the cardiovascular system may be more complex than initially conceived. Herein, we will review the significance of macrovascular abnormalities in the setting of CS and explore the growing role of microvascular tissue perfusion markers in disease management (Central Illustration). By identifying gaps and limitations in our current understanding, we aim to provide insights into potential avenues for refining treatment strategies and shifting toward a more nuanced, tissue-centric approach to CS management.

**Central Illustration:**
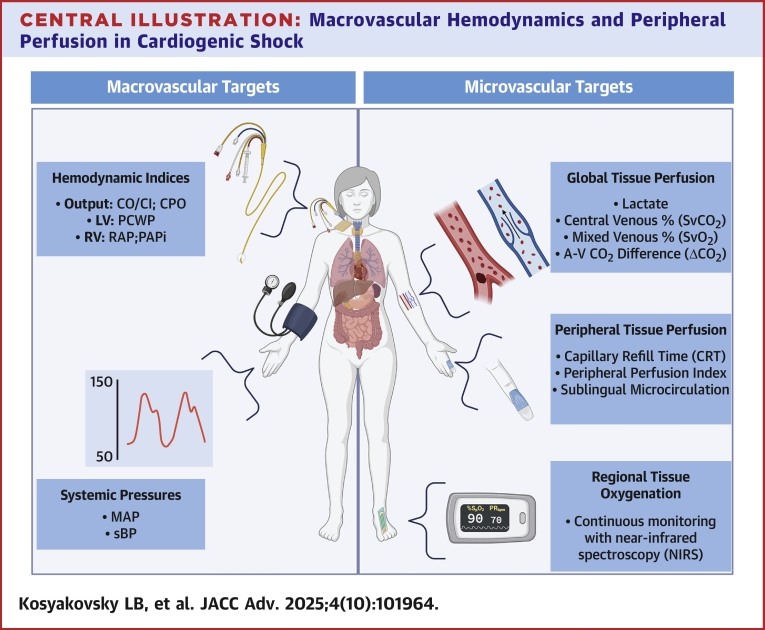
Macrovascular Hemodynamics and Peripheral Perfusion in Cardiogenic Shock Traditional macrovascular targets in cardiogenic shock include hemodynamic indices, such as cardiac output and filling pressures, and systemic pressures. Microvascular targets can offer important additional insight, including measures of global tissue perfusion, peripheral perfusion, and regional tissue oxygenation. CO = cardiac output; CPO = cardiac power output; LV = left ventricle; MAP = mean arterial pressure; PAPi = Pulmonary Artery Pulsatility Index; PCWP = pulmonary capillary wedge pressure; RAP = right atrial pressure; RV = right ventricle; sBP = systolic blood pressure.

## Macrovascular hemodynamic targets

### Systemic arterial blood pressure

One of the cornerstones of CS management is maintaining an optimal mean arterial pressure (MAP), but the appropriate range across different shock states remains uncertain. In general, targeting a MAP between 60-65 mm Hg and/or a systolic blood pressure (SBP) of ≥90 mm Hg is recommended for CS,^3^ though in patients with wide pulse pressure, these MAP and SBP targets can reflect substantial differences in perfusion. However, these differences may be driven by autoregulation, which operates through the contraction and dilation of upstream arterioles in response to increases and decreases in systemic pressure, respectively.^4^ As there is often a lower limit of blood pressure to which an arteriole can compensate with dilation, individuals with baseline hypertension may have different thresholds for inadequate perfusion, ranging between 50 and 150 mm Hg.

Much of the evidence for MAP targets in the management of CS has been extrapolated from other forms of circulatory shock. One trial found that in patients with septic shock, targeting a relatively lower MAP (65-70 mm Hg) achieved favorable outcomes—reinforcing the supposition that targeting a MAP of 60 to 65 mm Hg in patients with CS may be reasonable.^5^ Burstein and colleagues evaluated this hypothesis in a single-center, retrospective analysis. Among 1,002 patients with CS, they found that patients who survived to hospital discharge had a higher mean MAP during the first 24 hours of admission compared to nonsurvivors (75 mm Hg vs 71 mm Hg; *P* < 0.001)^6^ and that those with a mean 24-hour MAP <65 mm Hg were nearly twice as likely to die in-hospital compared to a MAP >65 mm Hg (57% vs 28%; adjusted OR: 2.0; *P* < 0.001). No difference in outcomes was observed when patients with a mean 24-hour MAP 65 to 74 mm Hg were compared to those with a mean 24-hour MAP ≥75 mm Hg (*P* > 0.10).^6^ These findings suggest that, while MAPs below 65 mm Hg are potentially harmful, targeting MAPs above 75 mm Hg provides no additional benefit. Meanwhile, in a post hoc analysis of the DOREMI (Milrinone as Compare with Dobutamine in the Treatment of Cardiogenic Shock) trial—a randomized, double-blind trial comparing dobutamine to milrinone in patients with CS—patients with a mean MAP ≥70 mm Hg (high MAP) in the first 36 hours following randomization were compared to those with a mean MAP <70 mm Hg (low MAP).^7^ The combined major adverse cardiac events (MACE) outcome occurred in 42.2% vs 67.6% of patients in the high and low MAP groups, respectively (adjusted relative risk: 0.70; *P* = 0.01). Additionally, lower all-cause mortality was observed in the high MAP (28.9%) compared to the low MAP (57.8%) cohorts (adjusted relative risk: 0.56; *P* < 0.01) (Table 1).

**Table 1:** Macrovascular Hemodynamic Targets

Studies First Author, Year	Size (n)	Study Type	Finding
Section A. Mean Arterial Pressure			
Asfar et al, 2014^5^	776	Multicenter double-blinded study of patients with septic shock (SEPSISPAM)	No significant difference in mortality between patients with low or high MAP
Burstein et al, 2020^6^	1,002	Single-center retrospective analysis of patients with CS	Patients who survived to discharge had a higher mean MAP during first 24 h Patients with mean 24 h MAP <65 mm Hg had almost 2X in-hospital mortality as those with MAP >65 mm Hg No mortality difference in patients with MAP 65-74 mm Hg vs those with MAP ≥75 mm Hg
Parlow et al, 2021^7^	192	Post hoc analysis of randomized double-blind trial comparing inotropes in patients with CS (DOREMI)	Lower all-cause mortality in patients with low MAP compared to patients with high MAP, and no significant difference in major adverse cardiovascular outcomes.
Section B. Cardiac Output (CO) and Pulmonary Capillary Wedge Pressure (PCWP)			
Hasdai et al, 1999^8^	2,698	Post hoc analysis of patients with CS in the Global Utilization of Streptokinase and Tissue Plasminogen Activator for Occluded Coronary Arteries (GUSTO-I)	Patients with CS and PCWP ≥20 mm Hg and CO ≥ 5.1 L/min had lower mortality rates
Section C. Cardiac Power Output			
Mendoza et al, 2007^9^	349	Prospective study of patients with acute cardiac disease admitted to Cardiac Care Unit undergoing PAC	Patients with CPO ≤0.53 W had significantly higher in-hospital mortality
Fincke, 2004^10^	541	Randomized prospective trial of patients with CS	CPO ≤0.53 W identified as strongest predictor of in-hospital mortality Every 0.2 W increase in CPO correlated in 45% decrease in odds of death
Garan et al, 2020^11^	1,414	Retrospective analysis of patients with CS	CPO did not predict mortality, but complete hemodynamic monitoring did
Basir et al, 2018^12^^,^^13^	41	Prospective study of patients with AMI and CS	Utilization of CPO >0.6 as a therapeutic target for treatment guidelines surrounding mechanical circulatory support and percutaneous coronary intervention was associated with improved survival to discharge. CPO <0.6 W was associated with decreased mortality when used as a part of a structured shock protocol.
Section D. Right Atrial Pressure			
Thayer et al, 2020^14^	1,414	Retrospective analysis of CS registry	Elevated RAP was associated with worse SCAI staging and high rates of in-hospital mortality
Section E. Pulmonary Artery Pulsatility Index			
Korabathina et al, 2012^15^	20	Prospective study of patients with acute inferior MI	PAPI is decreased in patients with right ventricular dysfunction and was the most sensitive and specific predictor for in-hospital mortality
Fried et al, 2018^16^	132	Retrospective review of CS patients with IABP	PAPI identified as most significant predictor of clinical deterioration on IABP
Tehrani, 2019^17^	204	Single-center prospective registry of CS patients	PAPI <1.0 at 24 h after diagnosis identified as an independent predictor of 30-d mortality

AMI = acute myocardial infarction; CPO = cardiac power output; CS = cardiogenic shock; IABP = intra-aortic balloon pump; MAP = mean arterial pressure; MI = myocardial infarction; PAC = pulmonary artery catheter; PAPI = pulmonary artery pulsatility index; RAP = right atrial pressure; SCAI = Society for Cardiovascular Angiography and Interventions.

However, there are still limited data regarding the utility of targeting a higher MAP. Additionally, the challenge of reaching adequate MAP targets in patients who are more ill may confound this association. Further complicating this issue is the occasional absence of overt hypotension in CS, as arterial pressure may be preserved in some patients with compensatory vasoconstriction with or without vasopressor agents.^18^

### Invasive hemodynamics

While invasive hemodynamic monitoring has been associated with reduced in-hospital mortality in patients with cardiogenic shock (CS), no prospective randomized trials have demonstrated a clear benefit in guiding care based on these parameters.^19^ A comprehensive review of these data has previously been published by Mathew et al and many of the key studies have been summarized in Table 1.^20^ Further studies, such as the ongoing PACCS (Pulmonary Artery Catheter in Cardiogenic Shock) trial (NCT05782780), are needed to establish the role of invasive hemodynamic monitoring in CS, including contributions to overall mortality as well as the restoration of macrovascular and/or microvascular perfusion.

## Clinical indices of peripheral perfusion

### Capillary refill time

Despite the development of precise invasive tools such as the arterial line and the pulmonary artery catheter, the quickest, cheapest, and simplest assessment of a patient’s peripheral perfusion continues to be the bedside physical examination, including techniques such as capillary refill time (CRT). CRT is defined as the time taken for the color to return to a peripheral capillary bed after external pressure has been sufficiently applied to result in blanching. Although a normal CRT has been variably defined since it was first formally applied in 1947, most clinicians deem a CRT of <2 to 3 seconds as normal.^21^

Among 61 patients admitted to the intensive care unit (ICU) with CS, a prolonged CRT of more than 3 seconds was associated with increased 90-day mortality or the need for venoarterial extracorporeal membrane oxygenation (ECMO) support (HR: 12.38; 95% CI: 2.91-52.71). Interestingly, CRT did not correlate with macrovascular parameters such as HR, SBP, cardiac index (CI), and capillary perfusion index, but increased CRT was significantly correlated with multiple microcirculatory parameters including mottling, urine output, and central venous-arterial carbon dioxide difference, indicating that CRT may provide critical insight into micro-, rather than macro-, circulatory dysfunction.^22^ While CRT has clinical utility as a marker of peripheral perfusion, CRT assessment may be limited by confounders such as age, temperature, ambient light, and pressure application as well as poor intraobserver and interobserver reliability.^23^ Thus, CRT is considered to be an adjunct means of assessment of perfusion rather than a gold standard.

### Peripheral perfusion index

An additional noninvasive measure of tissue perfusion is the peripheral perfusion index (PPI), defined as the ratio between the pulsatile and nonpulsatile component of the pulse oximetry waveform.^24^ Among critically ill patients with circulatory shock, there was a concordant linear correlation between the change in PPI and the core-to-toe temperature difference (R^2^ = 0.52; *P* < 0.001). A PPI of 1.4 discriminated best between a normal and abnormal core-to-toe temperature difference (area under the curve [AUC]: 0.91; 95% CI: 0.84-0.98). To assess the prognostic valve of PPI in critically ill patients, investigators measured PPI 8 hours after early resuscitation in 202 ICU patients. They found the greatest AUC (0.835) for the prediction of 30-day mortality, outperforming ScvO_2_, P(v-a)CO_2_, and lactate.^25^ The combination of low ScvO2 and low PPI was associated with the lowest survival rate at 30 days, suggesting that complementary ScvO2 and PPI assessment may better identify endpoints of resuscitation and adverse patient outcomes (Table 2). There was no significant relationship between PPI and macrovascular measures including MAP, supporting its unique role in microvascular assessment. A potential limitation with PPI is that it may be less accurate in patients with severe CS, particularly those treated with mechanical circulatory support, given decreased pulsatility. In addition, PPI interpretation can be substantially limited by patient factors, such as pain, body temperature, and peripheral vascular disease, in addition to clinical factors such as disease state, which introduce substantial variation in PPI skew and threshold.^37^

**Table 2:** Clinical Indices of Perfusion

Studies First Author, Year	Size (n)	Study Type	Finding
Section A. Clinical Capillary Refill Time (CRT)			
Lima et al, 2009^26^	22	Prospective study of ICU patients admitted with high lactate levels	Patients with increased CRT were more likely to have low tissue oxygen saturation, and higher rates of clinical complications
Lima et al, 2009^27^	50	Prospective study of ICU patients (only 2 of 50 patients admitted had CS)	Increased CRT identified patients who were hemodynamically stable but had higher lactate levels and more severe end organ dysfunction and more likely to have poor outcomes
Hernández et al, 2019^28^	420	RCT, multicenter among ICU patients with early septic shock	CRT-targeted resuscitation was associated with less organ dysfunction at 72 h, and there was a trend toward improved mortality compared with standard of care
Merdji et al, 2022^22^	50	Prospective study of ICU patients with CS	CRT values were associated with higher 90-d mortality rates, and higher rates of VA-ECMO utilization.
Hariri et al, 2024^29^	27	Retrospective study of ICU patients with CS on VA-ECMO	CRT decreased significantly in patients who responded to fluid resuscitation, suggesting it can be used as a reliable tool to assess fluid responsiveness
Section B. Peripheral Perfusion Index (PPI)			
Lima et al, 2002^24^	108 healthy adults 37 critically ill patients	Prospective study in healthy adults and ICU patients	In healthy adults, PPI varied in relation to mealtime. In critically ill patients, decreased PPI was found to be associated with other markers of peripheral perfusion (eg, core-to-toe temp difference)
Lima et al, 2009^27^	50	Prospective study in ICU patients	Subjective assessment of PPI was consistent with other clinical indicators of hemodynamic instability and could identify patients at risk of worsening organ failure
He et al, 2015^25^	202	Prospective study in patients with central venous catheters	Low PPI (≤0.6) and low SvO_2_ (<70%) were predictive of high 30-d mortality rates
Section C. Sublingual Microcirculation (SM)			
DeBacker et al, 2004^30^	40	Prospective study, nonblinded in patients with CS	CS decreases the number of perfused vessels detected, but the velocity of blood flow in perfused vessels remained similar to controls. Vascular density was only decreased in microcirculation not in macrocirculation Decreased SM was associated with increased lactate, decreased oxygen extraction, and increased mortality SM perfusion was not related to intra-aortic balloon pump (IABP) use, but acetylcholine application normalized microcirculation perfusion
Den Uil et al, 2010^31^	68	Prospective study in patients with CS	Diminished perfused SM density was a predictor of increased mortality in patients with CS
Den Uil et al, 2014^32^	30	Prospective study in patients with CS	Inotropes did not change sublingual perfused capillary density and patients with decreased perfused capillary densities had increased mortality rates compared to those with higher or preserved perfused capillary densities
Wijntjens et al, 2020^33^	66	Multicenter subanalysis of patients with CS complicated AMI (substudy of CULPRIT-SHOCK)	Normotensive patients with decreased microcirculatory perfusion parameters were more likely to experience all-cause death
Bruno et al, 2023^34^	141	Prospective multicenter trial, among patients with elevated lactate on vasopressors (approximately 50% with CS)	Use of sublingual microcirculatory perfusion variables in the therapy plan resulted in more treatment changes without a survival benefit
Section D. Regional Tissue Oxygenation			
Filho et al, 2020^35^	40	Prospective study of patients with circulatory shock	NIRS-derived parameters were the only measurement that distinguished between patients in CS and controls
Lima et al, 2009^26^	22	Prospective study of ICU patients admitted with high lactate levels	Patients with low StO_2_ levels following initial resuscitation had worse organ failure than patients with normal StO_2_ changes, but StO_2_ is unrelated to global hemodynamics.
Varis et al, 2020^36^	927	Systematic review of 18 observational studies of patients with circulatory shock in which NIRS was used to assess perfusion	StO_2_ calculated from NIRS measurements is a good predictor of mortality in patients with circulatory shock

AMI = acute myocardial infarction; ICU = intensive care unit; NIRS = near-infrared spectroscopy; RCT = randomized controlled trial; VA-ECMO = venoarterial extracorporeal membrane oxygenation.

### Sublingual microcirculation

Another method to quantitatively assess perfusion of microcirculation at the bedside is with direct visual observation of the capillary beds with videomicroscopy. Orthogonal polarization spectral (OPS) imaging is a noninvasive technique where polarized light illuminates the area of interest, reflected by the background, and then absorbed by hemoglobin, resulting in high-contrast images of microcirculation. Unfortunately, capillary visualization may be suboptimal, the images may blur due to the motion of red blood cells, and excessive probe pressure may also modify red blood cell velocity during OPS imaging. In contrast to OPS, Sidestream dark field (SDF) illumination is created by concentrically placed light-emitting diodes, which addresses some of the issues with OPS and provides improved image quality.^38^ Notably, both OPS and SDF devices have been largely relegated as research tools due to technological limitations which predispose them to poor reproducibility and lack of automatic quantification. These limitations led to the development of a third-generation device which uses incident dark field illumination (Cytocam-IDF), a pen-like probe with a sterilizable cap that serves as a handheld microscope where a high-density pixel-based imaging chip and short pulsed illumination source under computer control synchronizes and controls illumination and acquisition.^39^

Among 40 patients with acute severe heart failure, including acute decompensated heart failure (ADHF)-CS, OPS imaging was used to assess sublingual microcirculation, often considered to be an accessible surrogate for splanchnic microvasculature.^30^ Although the density of vessels was similar between cardiac failure, CS, and control patients, the proportion of perfused small (<20 ⴗm) vessels was lower in patients with cardiac failure and CS compared to controls (cardiac failure 63%; CS 49%; control 92%; *P* < 0.001). Additionally, the proportion of perfused capillaries (PPC) was higher in patients who survived (90% vs 81%; *P* < 0.05). Interestingly, the proportion of perfused small vessels was associated with lactate concentration and oxygen extraction, but not to MAP, SvO2, CI, systemic vascular resistance, arterial pH, or use of vasoactive agents.

One prospective study of 68 patients with acute myocardial infarction (AMI)-CS used SDF imaging to measure the sublingual perfused capillary density (PCD) and found that diminished sublingual PCD, both at baseline and following treatment, is associated with development of multi-organ failure and a predictor of poor clinical outcomes.^31^ A subsequent prospective study examining the effects of inotropic agents on tissue perfusion parameters in 30 patients with AMI-CS found that patients with low final PCD had a higher mortality rate compared to those with preserved PCD, irrespective of achieving macrohemodynamic optimization.^32^

Finally, in a predefined multicenter analysis of the CULPRIT-SHOCK (Culprit Lesion Only PCI Versus Multivessel PCI in Cardiogenic Shock) study, SDF and IDF technology were used in 66 patients presenting with AMI-CS.^33^ There was a significant adjusted association between PCD (HR: 0.947; *P* = 0.035) and PPC (HR: 0.986; *P* = 0.020) and the primary combined clinical endpoint of all-cause death and renal replacement therapy at 30 days, which was not identified for SBP (HR: 0.987; *P* = 0.203). Moreover, even among the 77.3% of patients who had a normal MAP, the primary endpoint was more frequent in patients with an abnormal PPC.

Systematic analyses of microvascular function have made it increasingly apparent that CS may represent a distinct uncoupling of macrovascular and microvascular circulation, and moreover, that monitoring perturbations of microvascular function may more reliably predict prognosis than traditional macrovascular measures in CS.^40^ As the physiologic gradient from macrovascular to microvascular to metabolic processes is further delineated, researchers have turned their attention to tissue perfusion as it relates to regional oxygenation, arguably one of the most important functions of the circulatory system.

### Regional tissue oxygenation

Near-infrared spectroscopy (NIRS) is an indirect imaging modality which functions on the principle that, in the near-infrared band of roughly 700 to 900 nm, different biological tissues absorb near-infrared light at different wave lengths. Deoxygenated and oxygenated hemoglobin can be differentiated in this manner, presenting the opportunity to continuously monitor regional tissue oxygenation (StO_2_). Tissue oxygenation is dependent upon the proportion of oxygenated hemoglobin concentration relative to total hemoglobin concentration, which is affected by contributions from small blood vessels (<1 mm in diameter) such as venules, arterioles, and capillary beds. Light is completely absorbed by blood vessels exceeding 1 mm in diameter and are thus excluded. NIRS data primarily reflects changes in the oxygenated hemoglobin concentration within the venous compartment, as this is where approximately 70% of the blood volume is located.^41^

Tissue oxygenation data from NIRS devices used in the operating room and critical care setting have predicted postoperative delirium, postoperative cognitive dysfunction, and extended ICU length of stays.^42^ NIRS-derived parameters can even discriminate between critically ill patients with and without circulatory shock, whereas other microcirculatory perfusion parameters such as CRT, PPI, and skin-temperature gradient could not.^35^ Furthermore, critically ill patients who continue to have low StO_2_ despite initial resuscitation develop significantly worse organ failure than those with normal StO_2_.^26^

There is conflicting evidence regarding whether StO_2_ changes correlate with macrovascular hemodynamic variables. While one study of critically ill patients suggested there is no correlation between changes in StO_2_ and macrohemodynamic parameters, another, the venoarterial-ECMO study, concluded that SctO_2_ and peripheral StO_2_ can be used as noninvasive surrogates of macrohemodynamic status such as MAP, CI, and systemic vascular resistance index.^26^^,^^43^ A systematic review of 18 observational studies that included 927 patients determined that, while NIRS-derived StO2 can predict mortality in circulatory shock, high-quality data on the impact of NIRS monitoring are lacking.^36^ To date, there are no data to clearly support an association between cerebral tissue oxygenation (SctO_2_) and outcomes in patients with CS, nor has the impact of inotrope therapy and mechanical support devices on SctO_2_ been evaluated (Table 2).

## Biochemical indices of peripheral perfusion

### Lactate

Multiple laboratory markers offer insight into the state of systemic perfusion in CS, of which lactate has been the most well-studied. Lactic acidosis is a signature feature of CS, with up to 74% of patients presenting with an elevated lactate on admission and 40% experiencing an admission lactate elevation ≥5 mmol/L.^44^^,^^45^ Lactate elevation in CS occurs via multiple distinct pathophysiologic mechanisms, including true hypoperfusion (type A) and mechanisms unrelated to global hypoperfusion (type B).^46^ Type A lactic acidosis in CS typically results from reduced CO and global hypoperfusion.^47^ However, lactate elevation in CS can also be the result of adrenergic stimulation of lactate production (either through catecholamines produced during shock or exogenous administration as vasopressor agents) or mitochondrial dysfunction, particularly with concomitant sepsis.^48^ Thus, while lactate elevation is often assumed to reflect inadequate perfusion in CS, it is critical to consider other potential contributing mechanisms, particularly in the presence of conflicting clinical markers of perfusion.

Regardless of mechanism, lactate elevation is a critical diagnostic and prognostic tool in CS. Both the severity of lactate elevation and the presence of concomitant acidosis are important prognostic markers, with a presenting lactate ≥5 mmol/L or a pH <7.2 providing additional predictive power for 30-day mortality beyond Society for Cardiovascular Angiography and Interventions (SCAI) stage.^49^ While useful as a one-time marker of shock severity at presentation, lactate elevation carries important prognostic value from baseline to at least 24 hours after shock onset.^50^ Additionally, while lactate elevation itself reflects the presence of hypoperfusion, lactate clearance may also reflect the restoration of perfusion and an improving state of shock. In keeping with this, the presence of either partial or complete lactate clearance in the first 24 hours of shock has been demonstrated to be superior to the assessment of lactate levels at individual time points for predicting short-term mortality.^44^^,^^51^^,^^52^ One analysis found that lactate clearance of 3.45% per hour in the first 8 hours was the optimal cutoff for predicting mortality.^52^ While another analysis found that lactate clearance at 24 hours was the strongest predictor of survival with an OR of 5.44 (95% CI: 2.14-13.8; *P* < 0.01).^44^ Particularly in the assessment of lactate clearance, arterial lactate may be superior to venous measurements, given that venous lactate may reflect regional tissue perfusion and the heterogeneity of microcirculation in states of shock.^53^ This heterogeneity in local tissue perfusion may also explain why venous and arterial lactate measurements are highly correlated but have poor agreement in multiple studies.^54^, ^55^, ^56^ To this end, arterial lactate clearance has been shown to be superior to either venous lactate clearance or individual time points of venous lactate in the prediction of short-term CS mortality; arterial lactate clearance should therefore be considered the gold standard of lactate assessment in CS^52^ (Table 3).

**Table 3:** Biochemical Indices of Perfusion

Studies First Author, Year	Size (n)	Study Type	Finding
Section A. Lactate			
Jentzer et al, 2022^49^	1814	Retrospective cohort study of patients with CS	Patients with a lactate ≥5 mmol/L or blood pH <7.2 had increased risk of 30-d mortality regardless of SCAI stage. These patients had approximately 2-fold higher adjusted 30-d mortality when compared to their counterparts.
Marbach et al, 2022^44^	82	Post hoc analysis of patients with randomized double-blind controlled trial comparing inotropes in treating CS (DOREMI)	At baseline survivors and nonsurvivors had similar lactate levels; however, nonsurvivors had consistently elevated lactate levels at all following time points (between 0 and 36 h following admission). Complete lactate clearance was the strongest predictor of survival at all time points. Renal replacement therapy did not affect the rate of complete lactate clearance.
Lindholm et al, 2020^50^	219	Secondary analysis of prospective multicenter study in patients CS (CardShock study)	Lactate from the time of admission through 24 h is predictive of 30-d mortality. 50% reduction in lactate at 6 h, 12 h, and 24 h after admission were predictive of decreased mortality. Serum lactate levels were predictive of mortality independent of other predictive variables (such as prehospital resuscitation)
Marbach et al, 2021^51^	-	Systematic review of studies comparing lactate clearance in patients with cardiogenic shock	Lactate clearance is significantly associated with mortality. Patients with increased lactate clearance at 6-8 h and 24 h after admission had increased rates of survival compared those with decreased lactate clearance. At 6-8 h, survivors had a mean percentage lactate clearance of 21.9% while nonsurvivors had a mean percentage lactate clearance of 0.6%. At 24 h, survivors had a mean percentage lactate clearance between 50 and 80% while nonsurvivors had a mean percentage lactate clearance between −9.7% and 70%.
Scolari et al, 2020^57^	43	Prospective study of lactate levels in patients with CS before and after treatment with MCS	Increased serum lactate levels at all time points and lactate clearance only after 6 h were associated with higher mortality rates. Serum lactate >1.55 mmol/L at 24 h was the best prognostic marker of 30-d mortality, and failure to improve lactate at 24 h was associated with a mortality rate of 100%.
Section B. Central Venous O_2_ (ScVO_2_)			
Gallet et al, 2012^58^	60	Prospective study of patients with acute decompensated heart failure requiring inotrope support	ScVO_2_ <60% is associated with an increased rate of major adverse cardiac events
Section C. Mixed Venous O_2_ (SvO_2_)			
Muir et al, 1970^59^	26	Prospective study of patients with AMI	SvO_2_ below 65-70% indicates significant hypoperfusion and reduced CO
Section D. Arteriovenous ΔCO_2_			
McDonald et al, 2021^60^	31	Retrospective review of CS patients requiring venoarterial ECMO	ΔCO_2_ >6 mm Hg is associated with increased mortality at 24 h as it was a predictor of failure to wean from ECMO. Increasing ΔCO_2_ was also associated with increased mortality as were increased lactate levels.
Liang et al, 2023^61^	40	Retrospective review of patients requiring Impella 5.5	Decreased rates of ΔCO_2_ at 5d post Impella implantation was associated with improved rates of extubation and may be used as a predictor of successful extubation.

Abbreviations as in Tables 1 and 2. MCS = mechanical circulatory support.

### Oxygen saturation and carbon dioxide gap

In addition to lactate, arteriovenous oxygen difference, central venous oxygen saturation (ScvO_2_), mixed venous oxygen saturation (SvO_2_), and arteriovenous CO_2_ gap (ΔCO2) have been identified as important markers of perfusion in CS. Arteriovenous differences in oxygen are central to understanding CS, as CO is an important factor in global oxygen delivery. In the setting of CS, global oxygen delivery is inadequate to meet the metabolic requirements of peripheral tissues.^62^ Accordingly, management of CS focuses on improving CO and global oxygen delivery.^63^ Both ScvO_2_ and SvO_2_ reflect systemic tissue oxygenation and oxygen extraction.^64^ While ScvO_2_ and SvO_2_ saturation are similar in healthy individuals and often used interchangeably, they can differ substantially in critically ill patients.^65^ Creamer et al assessed oxygen transport variables in 19 patients with CS and found that—in patients resuscitated with fluids and dobutamine (protocol dependent on pulmonary capillary wedge pressure)—SvO_2_ increased from 54% to 69%, leading the authors to conclude that therapies should be titrated to increases in SvO_2_.^66^ In cases of CS, the use of SvO_2_ to titrate interventions is a mainstay of treatment. Typically, values of SvO_2_ below 65% to 70% reflect conditions of clinically relevant hypoperfusion and have been associated with reduced CO.^59^ However, changes in SvO_2_ may also be related to changes in arterial oxygen saturation, hemoglobin concentration, and tissue oxygen demands.^64^ In the absence of access to SvO_2_, ScvO_2_ may be cautiously utilized; a ScvO_2_ <60% has been associated with MACE in patients with ADHF-CS.^58^ Additionally, the ΔCO_2_ may also reflect tissue perfusion. Venous CO_2_ is typically determined by aerobic production of CO_2_, as influenced by the metabolic rate. Normally, only a very small arteriovenous CO_2_ gap exists (<2-6 mm Hg) in the presence of regular blood washout; any stagnation is a direct result of reduced CO, and therefore, stagnation may reflect tissue hypoperfusion and reduction in CO.^67^ Clinically, ΔCO2 is an important marker of supply-demand mismatch in tissue perfusion, reflecting the adequacy of CI to support a given metabolic condition.^68^ In the CS literature, a ΔCO2 > 6 mm Hg has been associated with increased short-term mortality in patients requiring ECMO support.^60^ Similarly, a low ΔCO2 predicts successful extubation in patients requiring Impella 5.5 (Table 4).^61^

**Table 4:** Summaries of Indices of Interest and Their Associations With Cardiogenic Shock

Index of Interest	Description	Physiology	Association With CS	Summary
Macrohemodynamic Indices				
Mean arterial blood pressure (MAP)	Average blood pressure throughout one cardiac cycle including systole and diastole.	MAP is dictated primarily by CO and SVR, with the primary goal of maintaining tissue perfusion SVR is determined by vessel radius, which is moderated by the release of competing vasoactive compounds like endothelin and NO. Increased MAP causes more strain on vessel walls, leading to the release of NO, leading to downstream smooth muscle relaxation and vessel dilation. CO is dictated by myocardial contractility, preload and afterload, volume status, heart rate, and conduction. MAP is generally decreased in patients with CS due to decreased CO and increased SVR due to compensatory vasoconstriction in hypoxic tissues.	MAP is often targeted in CS with medical and mechanical interventions such as inotropes and ventricular unloading devices. However, there is growing evidence that CS may not necessarily occur with decreased MAP, as compensatory vasoconstriction may sufficiently normalize MAP values. Current recommendation: Target MAP between 60-65 mm Hg and/or SBP of ≥90 mm Hg	Target MAP ≥65 mm Hg, however MAP is unlikely to be the sole driving force of hemodynamic instability in CS
Cardiac output (CO) and pulmonary capillary wedge pressure (PCWP)	CO is the amount of blood pumped by the heart and is affected by the heart rate (HR) and the volume of each contraction (stroke volume [SV]). CO = heart rate x stroke volume PCWP is an indirect measure of left atrial pressure	CO is the mechanism by which the rest of the body is perfused, and it can be affected by changes in HR and SV. Dysfunction affecting CO has a high likelihood of inducing end-organ damage as tissues lose the primary driver of perfusion. PCWP is measured by advancement of a catheter into the pulmonary artery and inflation of a balloon at its tip, estimating the pressure in the left atrium	In CS the CO drops, which may be reflected as an increase in LA pressure, which would be picked up on PCWP.	Patients with CS and increased PCWP and CO had lower mortality rates
Cardiac power output (CPO)	CPO incorporates pressure and flow in order to express the cardiac pumping ability of the heart. It is calculated by the following equation: CPO = (MAP X CO)/451	Cardiac power is based on the rule of fluids which says that power = flow x pressure. Cardiac power logically increases during increases in cardiovascular output, such as exercise, and is an early indicator of cardiac failure, as is the case with CS.	CPO may be compromised in patients with CS due to decreases in both CO and possible decreases in MAP	CPO ≤0.53 W is associated with increased rates of in-hospital mortality, and CPO may be used as a noninvasive macrohemodynamic indicator for CS, however it should not be used in isolation
Right atrial pressure (RAP)	RAP is a less invasive measurement of right heart function, compared to other hemodynamic measurements, like PCWP.	Increased RAP is a sign of heart failure, indicating right ventricular dysfunction and likely systemic congestion.	Decreased CO as is seen in CS will classically lead to increased PCWP, RV pressure and consequently RAP. CS is most often associated with MI, which classically affect the LV, so an elevated RAP is often a late indicator of CS, so should be noted as an indicator or CS severity.	Elevated RAP is associated with more advanced SCAI stages and poor in-hospital mortality rates.
Pulmonary artery pulsatility index (PAPI)	Pulmonary artery pulsatility index is the ratio of pulmonary arterial pulse pressure to right atrial pressure, representative of RV function. PAPI = (systolic pulmonary artery pressure - diastolic pulmonary artery pressure)/RAP	PAPI is used as a surrogate marker for RV function	Low PAPI represents an increased RAP relative to pulmonary artery pressure. In CS, decreased CO leads to backup into the right heart causing elevated RAP.	PAPI <1.0 is a significant predictor of clinical deterioration and in-hospital mortality
Clinical indices				
Capillary refill time (CRT)	Capillary refill time is a physical exam technique that measures the time to recover from blanching of peripheral capillary bed (usually at finger) via external pressure. Normal CRT <2-3s	Increased CRT indicates decreased capillary perfusion, either due to decreased CO, increased SVR, or other hemodynamic	CS leads to microcirculation dysfunction, which may be evidenced by increased CRT as arteriolar constriction, decreased CO. Peripheral perfusion at extremities may be used as a proxy for end-organ hypoperfusion in CS	CRT may be a useful addition to the bedside CS assessment toolbox, as higher CRT is associated with higher lactate levels, more severe end-organ dysfunction and poor outcomes
Peripheral perfusion index	Peripheral perfusion index is the ratio between the pulsatile and nonpulsatile component of pulse oximetry probe	Decreased PPI suggests a smaller difference in pulsatile and nonpulsatile oxygen saturations as measured by the probe.	In CS, decreased PPI would reflect decreased oxygenation differences across capillary beds, suggesting there is no difference in oxygen saturation during systole and diastole where there should be.	Decreased PPI may be used in conjunction with other markers, specifically SvO_2_, however on its own it is not a specific predictor for mortality in patients with CS
Sublingual microcirculation	Sublingual microcirculation is measured by polarized light that allows for the visualization of hemoglobin within vessels near epithelial surface, such as mucosal microcirculation sublingually	Sublingual microcirculation is a representative bed of small vessels demonstrated to change disproportionally to changes in macrocirculation	CS decreases the number of small vessels present in peripheral tissues, including sublingual circulation	Sublingual microcirculation may be an effective herald of larger hemodynamic instability, and decreased SM is associated with increased mortality rates. SM is unchanged by inotropes or intra-aortic balloon pump use.
Regional tissue oxygenation	The regional tissue oxygenation is measured by near-infrared spectroscopy (NIRS) which asses the proportion of oxygenated to deoxygenated hemoglobin within the venous compartment	Measured oxygenation is dependent on the relative contribution of small vessels to large vessels as light is absorbed by larger vessels.	CS decreases forward flow out of the heart, and decreased tissue oxygenation, and NIRS theoretically is an ideal way to noninvasively assess microcirculatory perfusion	Although no studies have conclusively assessed NIRS monitoring in patients with CS, in patients with circulatory shock, NIRS is demonstrated to be predictive of mortality and unrelated to macrohemodynamics
Biochemical indices				
Lactate	Lactate is an organic acid produced during anaerobic metabolism	Under normal conditions, lactate is produced by cells that can only perform anaerobic metabolism, such as skeletal muscle, brain tissue, and red blood cells. Lactate levels rise in states of hypoperfusion (type A) as tissues that perform aerobic metabolism switch to anaerobic metabolism. Lactate levels may also rise in nonhypoperfusive states (type B), such as sepsis, mitochondrial dysfunction, and with adrenergic stimulation	Patients with CS exhibit a wide spectrum of hemodynamic compromise, leading to end-organ hypoperfusion and a switch in tissue metabolism from aerobic to anaerobic mechanisms, increasing lactate production. Additionally, CS leads to the sustained adrenergic stimulation, further increasing lactate release. Lactate clearance is also affected by CS as its utilization in gluconeogenesis is inhibited in acidotic states, and it is dependent on hepatic function, which is often compromised in CS patients with significant comorbidities.	Changes in lactate levels has been identified as an indicator of hemodynamic instability and end-organ hypoperfusion as tissues are forced to change to anaerobic metabolism to meet energy demands. Patients with decreased lactate clearance and higher levels of serum lactate are at higher risk of mortality.
Central venous O_2_ (ScVO_2_)	Central venous O_2_ is the O_2_ saturation of venous mixture from upper body only. Measured with catheter in SVC, less invasive than SvO_2_.	ScVO_2_ represents regional oxygen consumption from upper limb and cranial circulation only. ScVO_2_ reflects venous flow from SVC, which includes jugular and subclavian venous supplies, as well as azygous and hemiazygos return.	In CS, tissue O_2_ extraction increases as O_2_ demands increase and supply decreases. This causes venous oxygen saturation to globally decrease as more O_2_ is pulled into tissues.	While ScVO_2_ is less invasive than SvO_2_, it also presents an incomplete image of venous hemodynamics in CS.
Mixed venous O_2_ (SvO_2_)	Mixed venous O_2_ is the O_2_ saturation of venous mixture from upper, lower, and cranial circulation. Measured with pulmonary artery catheterization	SvO_2_ reflects venous return from entire body, including lower limb, coronary and hepatosplanchnic circulation.	CS often leads to hypoperfusion in the hepatosplanchnic circulation, which is only captured by SvO_2_ measurements, not by ScVO_2_. Thus SvO_2_ is preferred in CS.	While ScVO_2_ is less invasive than SvO_2_, it also presents an incomplete image of venous hemodynamics in CS.
Arteriovenous (ΔCO_2_)	ΔCO_2_ is a measure of the difference in CO_2_ between the arterial and venous circulations, usually across a capillary bed, and under normal conditions, is equal to 2-6 mm Hg.	ΔCO_2_ can be used as an indicator of tissue perfusion and CO, as decreases in blood flow will cause an increase in CO_2_ release from tissues as they change from aerobic metabolism to anaerobic metabolism. CO therefore has an inverse relationship with ΔCO_2_.	In CS, decreases in CO will cause an increase in ΔCO_2_, which may be further compounded by tissue ischemia, lactate release, and acidosis.	High ΔCO_2_ is an indicator of flow stagnancy, suggesting low CO and in the setting of CS, is associated with poor outcomes. ΔCO_2_ may also be affected by metabolic compromise.

LA = left atrium; LV = left ventricle; RV = right ventricle; SBP = systolic blood pressure; SVR = systemic vascular resistance; other abbreviations as in Table 1.

### Discordant macrovascular hemodynamics and peripheral perfusion

In healthy individuals, a strong correlation has been noted between macrovascular hemodynamic indices (ie, MAP, CO, etc) and peripheral tissue perfusion. However, this correlation may become uncoupled in the presence of a pathological state such as CS. Therefore, while management efforts focused on normalizing macrovascular hemodynamics are unquestionably important, they may fall short when used in isolation to manage the microcirculatory and metabolic consequences of CS. As reviewed, the increasing body of work regarding the prognostic utility of markers of microvascular perfusion highlights the need for further evaluation of: 1) the discordance between macrovascular and microvascular indices in CS; and 2) the role for markers of microvascular perfusion in guiding therapies.

While the macrovascular hemodynamic insults of insufficient CO and global hypotension may precipitate the clinical manifestations of CS, the microvascular responses (ie, shunting of blood from capillary beds through metarterioles) and the resulting biochemical milieu of inadequate tissue perfusion may drive sustained decompensation. Constructing a framework for addressing the pathophysiological challenges in CS through a more microscopic lens may introduce complication, as each patient represents a heterogeneous mixture of microcirculatory insults, and different organ systems (ie, splanchnic vs skeletal tissue) respond differently to inflammatory and hypoperfused conditions. Nevertheless, the treating clinician must acknowledge and understand these challenges to more consistently restore adequate perfusion in patients with CS and improve clinically meaningful outcomes.

Based on the evidence outlined in this review, we believe that a contemporary approach to the management of CS, which emphasizes the importance of both pressure and flow, will be crucial in improving patient outcomes in the coming years. To achieve this goal, the evolution of CS management will require a deeper understanding of the processes that drive macrovascular and microvascular uncoupling in the patient with CS.

### Call for future investigation

Despite multiple innovations in the treatment of patients with CS over the past decades, the survival rate remains poor. One of the greatest challenges in CS management is the dearth of evidence specific to CS itself, as much of the data used to justify current clinical practice are extrapolated from a heterogeneous population of critically ill patients with circulatory shock. Thus, it is imperative that future investigations specifically study CS and systematically omit other forms of circulatory shock such as those individuals with sepsis, hemorrhage, or postoperative vasoplegia to enable more direct conclusions about patients with CS. One such promising effort is the CS working group, a multicenter registry for patients with CS, created to assess clinical outcomes within this population.

Given that patients with AMI-CS and ADHF-CS demonstrate different macrohemodynamic profiles, which in turn respond differently to established interventions, it may also be the case that the microhemodynamic adaptations in AMI-CS and ADHF-CS may also be unique. Future studies focusing on these important distinctions are essential to effectively extrapolate new data to our individual patients.

Lastly, as future studies focused on CS evolve, identifying the classification of the severity of CS within each cohort will likely prove to be an invaluable tool in applying the results to patients at the bedside. Existing efforts made by the SCAI to create and update a unified and standardized nomenclature for the classification of stages of CS will undoubtedly assist scientists and clinicians alike in tailoring study protocols, and ultimately, treatments of patients with CS. To further assist in the enhancement of our application of study protocols to the management of CS, we advocate for the use of invasive hemodynamic monitoring to better characterize and monitor dynamic macrohemodynamic profiles of this complex patient population.

In summary, we anticipate areas of highest impact in examining CS outcomes will require: 1) intentionally focusing on CS while systematically excluding other forms of circulatory shock; 2) specifying the subtype of CS based upon the instigating etiology; 3) providing continuous macrohemodynamic profiles obtained with invasive monitoring to better delineate various phenotypes; and 4) classifying the severity of CS based upon a unified and standardized nomenclature such as those provided by SCAI SHOCK Stage Classification. This standardized approach will help investigators to advance the field of CS management toward improving patient outcomes. Future investigations may examine whether targeting specific or combined macrovascular and microvascular metrics may best optimize tissue perfusion and guide medical and invasive therapy.

## Conclusions

The prevailing approach to CS management leans heavily on interventions directed at optimizing macrovascular hemodynamic indices, the targets for which have largely been extrapolated from a heterogeneous patient population. Emerging evidence summarized in this review suggests that CS may represent an uncoupling of macrovascular and microvascular hemodynamics. As such, certain parameters which more directly assess microvascular perfusion may serve as powerful prognostic indicators for patients with CS. Whether targeted therapies to improve microvascular perfusion will lead to meaningful improvement in clinical outcomes requires further investigation.

## Funding support and author disclosures

The authors have reported that they have no relationships relevant to the contents of this paper to disclose.
